# A Novel, Live-Attenuated Vesicular Stomatitis Virus Vector Displaying Conformationally Intact, Functional HIV-1 Envelope Trimers That Elicits Potent Cellular and Humoral Responses in Mice

**DOI:** 10.1371/journal.pone.0106597

**Published:** 2014-09-12

**Authors:** Svetlana Rabinovich, Rebecca L. R. Powell, Ross W. B. Lindsay, Maoli Yuan, Alexei Carpov, Aaron Wilson, Mary Lopez, John W. Coleman, Denise Wagner, Palka Sharma, Marina Kemelman, Kevin J. Wright, John P. Seabrook, Heather Arendt, Jennifer Martinez, Joanne DeStefano, Maria J. Chiuchiolo, Christopher L. Parks

**Affiliations:** 1 International AIDS Vaccine Initiative, Design and Development Laboratory, Brooklyn, New York, United States of America; 2 Molecular and Cellular Biology Program, The School of Graduate Studies, State University of New York Downstate Medical Center, Brooklyn, New York, United States of America; University of Massachusetts Medical Center, United States of America

## Abstract

Though vaccination with live-attenuated SIV provides the greatest protection from progressive disease caused by SIV challenge in rhesus macaques, attenuated HIV presents safety concerns as a vaccine; therefore, live viral vectors carrying HIV immunogens must be considered. We have designed a replication-competent vesicular stomatitis virus (VSV) displaying immunogenic HIV-1 Env trimers and attenuating quantities of the native surface glycoprotein (G). The clade B Env immunogen is an Env-VSV G hybrid (EnvG) in which the transmembrane and cytoplasmic tail regions are derived from G. Relocation of the G gene to the 5′terminus of the genome and insertion of EnvG into the natural G position induced a ∼1 log reduction in surface G, significant growth attenuation compared to wild-type, and incorporation of abundant EnvG. Western blot analysis indicated that ∼75% of incorporated EnvG was a mature proteolytically processed form. Flow cytometry showed that surface EnvG bound various conformationally- and trimer-specific antibodies (Abs), and *in-vitro* growth assays on CD4+CCR5+ cells demonstrated EnvG functionality. Neither intranasal (IN) or intramuscular (IM) administration in mice induced any observable pathology and all regimens tested generated potent Env-specific ELISA titers of 10^4^–10^5^, with an IM VSV prime/IN VSV boost regimen eliciting the highest binding and neutralizing Ab titers. Significant quantities of Env-specific CD4+ T cells were also detected, which were augmented as much as 70-fold by priming with IM electroporated plasmids encoding EnvG and IL-12. These data suggest that our novel vector can achieve balanced safety and immunogenicity and should be considered as an HIV vaccine platform.

## Introduction

More than 25 million people have died of AIDS since 1981 and an estimated 33 million people are currently living with Human Immunodeficiency Virus-1 (HIV-1) [1]. An effective vaccine remains the best option for ending the HIV pandemic. Models predict that even a partially effective vaccine introduced in high-risk countries could dramatically affect the number of new infections. For example, a vaccine with 50% efficacy administered to 30% of the general population would avert more than 5 million infections over 10 years, on top of any effect due to other preventative strategies [2] [3]. Live-attenuated simian immunodeficiency virus (SIV) vaccines have provided the most effective protection from progressive disease caused by homologous SIV infection of Rhesus macaques [4]–[6]; however, vaccines based on attenuated HIV-1 present too great a safety concern as a potential human vaccine. Attempts to develop a vaccine from inactivated HIV particles have failed, likely due to multiple factors such as poor growth and incorporation of the Envelope (Env) at low densities [7]–[11]. Therefore, other vaccine strategies, such as recombinant viral vectors carrying HIV immunogens, must be considered. Currently, replicating viral vectors are being tested as potential HIV-1 vaccine candidates because they can efficiently deliver vaccine immunogens in the context of a viral infection and have the potential to elicit cellular and humoral responses [12]. Moreover, the efficacy of live attenuated viral vaccines that protect from diseases such as measles, mumps, rubella, and varicella [13]–[15] provide a compelling rationale for developing an HIV vaccine based on a replicating vector.

**Figure 1:**
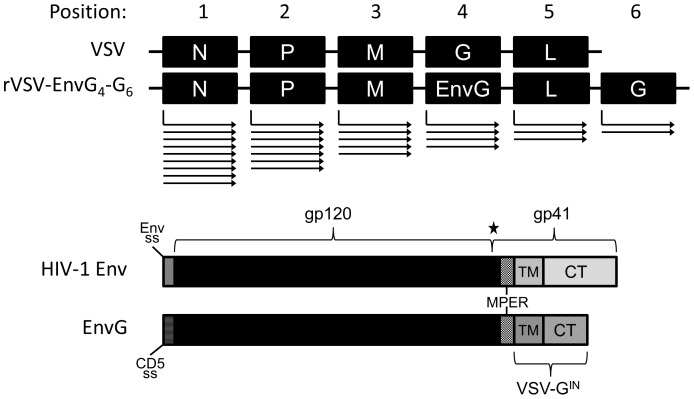
Genetic layout of the rVSV-EnvG_4_-G_6_ vector, HIV-1 Env, and the EnvG insert. VSV genes are shown in the 3′ to 5′ orientation as ordered on the recombinant genomic VSV plasmid and are not to scale. Arrows below each VSV gene depict the diminishing 3′-to-5′ mRNA transcription gradient. ss: signal sequence. MPER: membrane proximal external region. TM: transmembrane domain. CT: cytoplasmic tail domain. Star denotes site of intracellular Env(G) cleavage by furin.

Vesicular stomatitis virus (VSV) is particularly suitable for vaccine vector development. It infects multiple cell types, expresses foreign proteins abundantly, is highly immunogenic, and is not known to undergo homologous recombination, which is an important consideration for vaccine safety [16]–[18]. The viral genes are arranged in the VSV negative-sense RNA genome in the order 3′- *n* (Nucleocapsid)- *p* (Polymerase)- *m* (Matrix)- *g* (Surface Glycoprotein)- *l* (Large Protein)-5′, commonly known as the 5 ‘positions’ of the VSV genome due to its positional transcription gradient (Fig. 1). Transcription of the 5 mRNAs initiates from a single promoter at the 3′ terminus of the genome. Following transcription of *n*, intergenic regions control mRNA synthesis of the subsequent genes through a process of transcription termination and reinitiation. As reinitiation is not 100% efficient, a 3′ – 5′ transcription gradient is created with genes in the most 3′ ‘positions’ ultimately generating relatively abundant amounts of protein [19]. This feature of the VSV genome makes it a desirable candidate as a recombinant vaccine vector as it can be used to control gene expression and attenuate replication [18]. Immunogenicity of candidate recombinant VSV (rVSV) vectors has been confirmed in preclinical studies with a substantial range of immunogens derived from pathogens including influenza virus, Hepatitis C, and Ebola virus. Furthermore, although human infection with VSV is uncommon and is not associated with serious illness, people infected with the virus do produce antibodies (Abs) [18], [20]–[22]. Currently, highly attenuated VSV encoding HIV Gag is undergoing clinical assessment as a vaccine vector (ClinicalTrials.gov Identifier: NCT01438606) [23], [24]. Finally, VSV particles are known to incorporate foreign glycoproteins including Env during budding, which was advantageous for our research on live vectors designed to induce Abs against Env trimers [25]–[27].

Like HIV, VSV naturally enters through mucosal surfaces and infection presents the surface protein (G) as a trimer on infected cells and progeny virus particles [16]–[18]. Previous work by Rose, et al. demonstrated significant protection from SHIV 89.6P challenge after rhesus macaques (RMs) were immunized with rVSV vectors encoding SIV Gag and HIV-1 Env, with control of SHIV replication in vaccinated animals extending beyond 5 years [18], [28], [29] The rVSVs employed in this pioneering study were designed with either *env* or *gag* inserted after G, in the 5^th^ position (rVSV-G_4_-Gag_5_/EnvG_5_; subscript numbers denote genomic positions as defined in Fig. 1), minimally disturbing the native gene configuration [19], [30], [31]. Though no pathogenesis was associated with inoculating macaques with rVSV in the Rose et al. study, intranasal (IN) inoculation of BALB/c mice with rVSV-G_4_-EnvG_5_ induced a 5%–15% loss of initial body weight, and intracranial inoculation of a similar rVSV (rVSV-Gag_1_-G_4_) was shown to induce significant neurovirulence (NV) and mortality [18], [28]. Furthermore, in a NV study conducted with cynomolgus macaques, 1 of 4 animals inoculated intrathalmically with rVSV-G_4_-Gag_5_ demonstrated significant NV similar to that resulting from wild-type (WT) VSV [18], [32].

Following from previous work with rVSV-HIV vectors, our objective was to develop a genetically-stable live rVSV vector that would deliver abundant and authentic Env trimers that closely imitated functional membrane-bound trimeric spikes. We are pursuing this live vector design because trimeric Env spikes are the only known targets of HIV neutralizing Abs (nAbs), and to date, broadly nAbs have been induced only in HIV-infected patients exposed to Env trimers on infected cells and progeny HIV particles [33]–[35]. To better imitate progeny HIV particles and potentially improve Env immunogenicity, we designed our vectors to take advantage of the fact that VSV can incorporate Env during the budding process [16], [17], [36], [37], particularly when the Env cytoplasmic tail (CT) is deleted.

Here we describe investigation of several Env and VSV modifications to determine if genetically stable rVSV vectors can be engineered with minimal disruption to the potent gene expression apparatus that are safe to administer, incorporate relatively high quantities of Env spikes into virus particles, and express reduced but functional quantities of VSV G. Our rVSV vector described here encodes a functional Env-G (EnvG; Fig. 1) chimeric polypeptide composed of the HIV-1 clade B (JR-FL) Env ectodomain and membrane proximal external region (MPER) joined to the VSV G transmembrane (TM) and (CT) domains from a gene cloned into the 4^th^ position of the VSV genome in place of G. Native G expression and virus replication has been diminished by placement of the G gene at the 6^th^ and final position of the VSV genome, previously postulated to be an attenuating translocation [18], [38].

## Materials and Methods

### Molecular cloning

The rVSV-G_6_ vectors (Fig. 1) were produced using a VSV genomic clone based on the nucleotide sequence of the Indiana serotype, Mudd-Summers strain (Genbank EF197793, [39]). Genes encoding either G from the Indiana serotype (G^IN^, Genbank EF197793) or New Jersey serotype (G^NJ^; Genbank V01214.1, [40]) were placed in the 6^th^ position in genomic VSV plasmid with coding sequence for EnvG inserted in position 4 normally occupied by G (Fig. 1). Briefly, the genomic clone was designed from the genebank file by introducing limited nucleotide substitutions to create unique restriction endonuclease cleavage sites, which were used to insert the EnvG gene in genomic position 4 under control of the natural G stop and start signals. Similarly, endonuclease cleavage sites were created to insert G downstream of L in the 6^th^ genomic position. G transcriptional control signals were derived from the M/G intergenic region and the L stop signal. The nucleotide sequence of modified VSV-EnvG_4_-G_6_ genomic clones were confirmed by sequencing.

To produce an HIV env gene optimized for inclusion in VSV, the HIV-1 Env JR-FL protein sequence was backtranslated from GenBank sequence AAB05604.1. The Optimizer Web Tool [41] was used to backtranslate the Env amino acid sequence using a codon bias [42] typical for VSV generating a synthetic coding sequence. To enhance Env surface expression and incorporation into VSV particles, the Env TM and CT domains were replaced with those of VSV *g*, creating the *envG* gene [16], [36]. The Env signal peptide also was replaced with signal sequence (SS) from VSV G or cellular CD5 signal sequence [43]. As a precaution, gene sequences that resembled potential splice donor and acceptor signals were identified and removed by using synonymous codons [44]. In order to eliminate mutation-prone hotspots we have observed with VSV vectors, synonymous codons were used to interrupt stretches of homopolymeric stretches that were greater than 4 nucleotides in length. Finally, any sequence similar to an AU-rich element known to promote mRNA instability [45] was disrupted with synonymous codons and a Kozak translation initiation sequence [46] was included. GenScript (Piscataway, NJ) synthesized the EnvG gene.

An EnvG coding sequence also was generated for use in plasmid DNA vaccines by optimizing the sequence for expression in mammalian cells using the tools described above and a codon bias typical of mammalian cells. The optimized gene was synthesized by Genscript and cloned into the pCMV-kan [47] expression vector (kindly provided by B. Felber and G. Pavlakis, NCI). B. Felber and G. Pavlakis also provided murine pIL-12 (plasmid AG250, [48]). DNA used for vaccination was produced and purified by Aldevron LLC (Fargo, ND).

### Cell culture of recombinant VSV and analysis of gene expression

Vero or 293T cells were propagated in complete DMEM, which was Dulbecco's Modified Eagle media (DMEM; Life Technologies) supplemented with components from Gibco Life Technologies including 10% heat-inactivated fetal bovine serum, 1% sodium pyruvate, 1% L-Glutamine, and 1% Penicillin-Streptomycin and 220 µM 2-β-Mercaptoethanol. Monolayers were subcultured routinely following brief treatment with trypsin (Life Technologies).

VSV rescue from cloned DNA was performed using procedures described before except that plasmids were introduced into Vero cells by microporation [49]–[51]. Microporation was conducted using a BTX ECM 830 Electroporator device (Harvard Apparatus, Holliston, MA) with 4 mm gap electroporation cuvettes (VWR) with plasmids encoding each VSV structural protein (N, P, L, M, and G), a VSV genomic clone, and a plasmid encoding T7 phage RNA polymerase. Once cytopathic effect (CPE) was evident in the microporated cultures, virus in the medium supernatant was used to infect a fresh Vero cell monolayer and produce a working virus stock that was stored at −80°C. Two rounds of plaque purification were then performed to derive clonal isolates. Briefly, ten-fold dilutions of virus stocks were used to infect confluent Vero cell monolayers in 6-well plates after which they were overlaid complete DMEM containing 0.8% SeaKem LE agarose (Lonza). When plaques were visible, agar plugs were harvested and stored in 1 ml of DMEM, which was used for a subsequent round of plaque purification.

Infectious VSV was quantified by plaque assay. Near-confluent Vero cells monolayers were infected with serially diluted virus before being overlayed with medium containing agarose as described above. When plaques were visible, cells were fixed with 7% formaldehyde and stained with a solution of 20% crystal violet in water. Plaques were counted and infectious titers were expressed as plaque-forming units per milliliter (PFU per ml). An average titer from 3 wells was used to calculate PFU per ml.

Recombinant VSV vectors used for animal immunizations were propagated in Vero cell monolayers cultured in Viral Production Serum-Free Medium (VPSFM; Life Technologies) supplemented with 2% L-Glutamine and 1% Pen-Strep. Virus in clarified culture supernatants from infected cells was overlaid on cushions of 20% sucrose prepared in PBS, which were centrifuged for 1.5 hrs at 35,000 g at 4°C using a SW36 rotor (Beckman Coulter, Brea, CA). Viral pellets were resuspended in cold PBS and purified further using a 20% to 60% discontinuous gradient (40,000 rpm for 2 hrs at 4°C using a SW41 rotor; Beckman Coulter). Viral bands were harvested, pooled, and diluted 1∶5 in cold PBS before conducting a final concentration step by ultracentrifugation through sucrose cushions as described above. The virus pellets were resuspended in sucrose phosphate glutamate buffer (SPG [52]) buffer, aliquoted, and stored at −80°C. Before use in vaccination studies, PFUs were quantified, genomic sequencing was conducted, and Env expression in infected Vero cells was confirmed.

**Figure 2:**
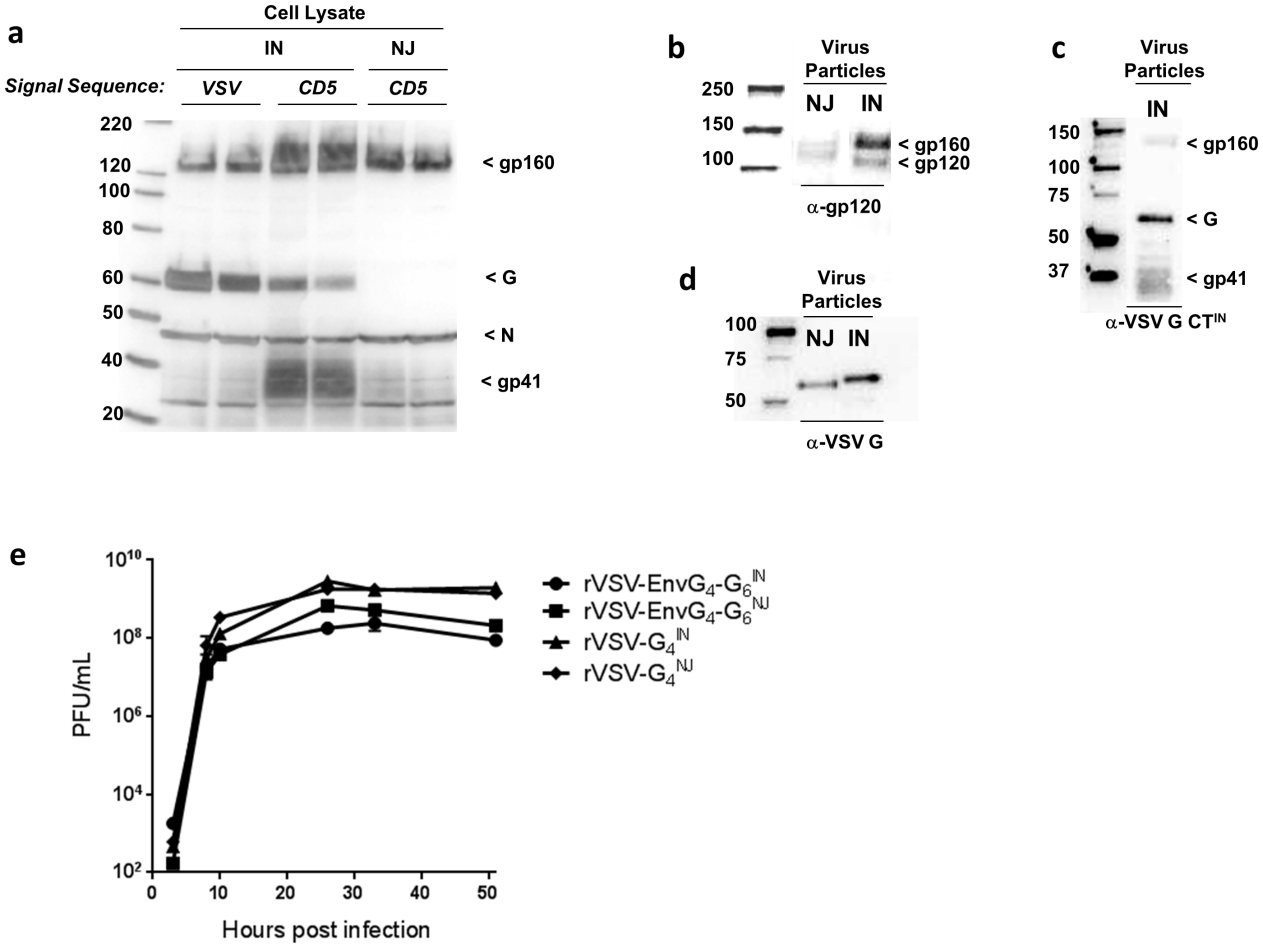
rVSV-EnvG_4_-G_6_ characterization. (a) After a 24 hr infection, total infected Vero cell lysates were collected and proteins were separated by SDS polyacrylamide gel electrophoresis (SDS-PAGE). Blot was probed with anti-VSV-N and anti-VSV-G_IN_ CT, which does not recognize G_NJ_. IN: rVSV-EnvG_4_-G_6_^IN^; NJ: rVSV-EnvG_4_-G_6_^NJ^. (b-d) Sucrose-gradient purified rVSV-EnvG_4_-G_6_ particles were separated by SDS-PAGE. (b) Blots of 10^6^ pfu of rVSV-EnvG_4_-G_6_^IN^ and 2.5×10^6^ pfu rVSV-EnvG_4_-G_6_^NJ^ vectors were probed separately for EnvG using anti-gp120. (c) Blot of 10^6^ pfu rVSV-EnvG_4_-G_6_^IN^ was probed with anti-VSV-G^IN^ CT. (d) Blot of 10^6^ pfu rVSV-EnvG_4_-G_6_^IN^ and rVSV-EnvG_4_-G_6_^NJ^ was probed with anti-VSV-G. (e) Replication kinetics of rVSV-EnvG_4_-G_6_ and rVSV-G_4_ viruses in Vero cells. Vero cells were infected in 6-well plates at an MOI of 0.1. At various intervals post infection, supernatant was collected from duplicate wells and virus was titrated. Plaques were counted manually after cell fixation.

**Figure 3:**
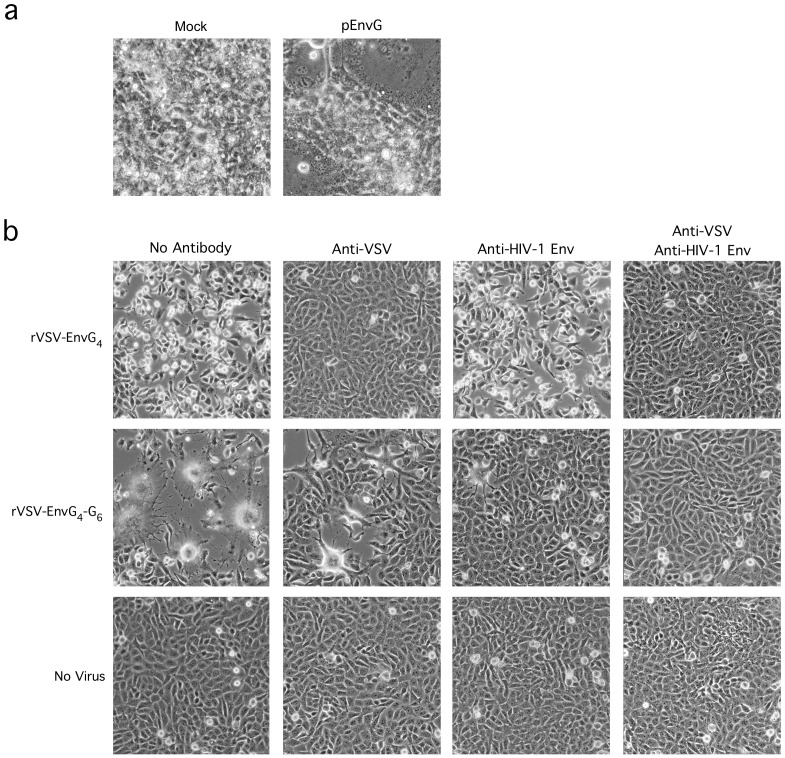
Fusogenicity and Functionality of EnvG. (a) 10^7^ 293T cells were transfected with pEnvG or empty vector using Mirus Trans-IT 293 according to manufacturer's protocol. 48 h post-transfection, 293T cells were overlaid with 2×10^6^ CD4+CCR5+ GHOST cells. 48 h after overlay, cells were visualized under light microscope and images were captured. (b) 10^6^ CD4+CCR5+ GHOST cells were infected as above after pre-incubation with anti-VSV-G (Vi10) and/or anti-Env cocktail. After infection, Vi10 and/or anti-Env cocktail was included in the culture media. Eight hours post infection, cells were visualized under light microscope and images were captured.

To study virus replication kinetics and attachment protein function, Vero or CD4+CCR5+ GHOST cells (Human osteosarcoma (HOS) cell line stably transformed to express human CD4 and CCR5 (HOS-CD4-CCR5), NIH AIDS Reagent Program, Division of AIDS, NIAID, NIH: Cat # 3318) were infected for 1 h at 0.1 PFUs per ml with rVSV-EnvG_4_-G_6_ or VSV lacking a foreign insert (rVSV-G_4_) [53], [54]. For Vero cell experiments, samples of medium supernatants were harvested at times specified in Figure 2e and virus was quantified by plaque titration as described above. For certain CD4+CCR5+ GHOST cell experiments (Fig. 3), virus was incubated with 100 ug/mL of anti-VSV-G Ab (Vi10) and/or 100 ug/mL each of the anti-HIV-1-Env nAbs PGT126, PGT128, PGT135, 2G12, and PGV04 (anti-Env cocktail) prior to infection [33], [55]–[57]. After infection, 100 ug/mL Vi10 and/or 100 ug/mL anti-Env cocktail was included in the culture media. Eight hours post infection, cells were visualized under light microscope and images were captured. All CD4+CCR5+ GHOST cells were cultured in the presence of 1 mg/ml puromycin, 0.5 mg/mL geneticin, and 0.1 mg/mL hygromycin (Life Technologies) to enforce the maintenance of the foreign genes.

Env expression was analyzed by infecting Vero cells with rVSV or by transfecting 293T cells. Vero cells were infected as described above. For pCMV-EnvG expression analysis, 293T cells were transfected with plasmid DNA using Lipofectamine (Invitrogen) or Mirus Trans-IT 293 (Fisher Scientific, Pittsburgh, PA), according to manufacturers' protocols. For membrane fusion experiments (Fig. 3), transfected 293T cells were overlaid with 2×10^6^ CD4+CCR5+ GHOST cells 48 h post-transfection, and images were captured as above 48 h after overlay. To assess protein expression, cell lysates were prepared from transfected 293T or infected Vero cells using CelLytic M buffer (Sigma, St. Louis, MO) supplemented with protease inhibitors (HALT Protease Inhibitor cocktail; Thermo Scientific) and incubated for 5 min on ice. Cell lysates were subjected to SDS polyacrylamide gel electrophoresis (SDS-PAGE) and subsequent Western blot analysis to detect EnvG using anti-gp120 (CA13; Center For AIDS Reagents, Hertfordshire, UK) or 0.2 ug/mL anti-VSV G^IN^ C-tail Ab (Sigma). VSV-G was detected with 1 ug/mL anti-VSV-G (Abcam), which reacts with G^IN^ or G^NJ^, or 0.2 ug/mL anti-VSV-G^IN^ CT Ab (Sigma). Band densitometry was quantified using Quantity One Version 4.6.3 (Bio-Rad, Hercules, CA). Western blot analysis also was performed with purified virus particles using about10^7^ pfu of virus per lane of a denaturing acrylamide gel.

### Cell surface and virus particle antibody staining

Infected Vero cells also were analyzed by flow cytometry. Monolayers were infected with 0.1 pfu per cell and incubated overnight at 37°. The following day, monolayers were washed with PBS and then treated with Cell Dissociation Buffer (Life Sciences) to produce a cell suspension. The cells were collected by centrifugation and resuspended before analysis with anti-VSV-G or anti-HIV-1 Env Abs as described previously [58].

To analyze Env incorporation in VSV particles, 10^9^ pfu of purified virus was bound to 100 ug Alum (Adju-Phos, Brenntag, Denmark) at 37°C with agitation for 30 min using methods adapted from those used before to analyze protein bound to Alum [59]. The rVSV/alum complexes were collected by centrifugation at 2000 rpm for 5 min., resuspended in blocking solution (3%BSA in PBS), and incubated at 37°C for 30 min with agitation. Complexes were collected by centrifugation and resuspended in 1% BSA prepared in PBS before aliquots of ∼5×10^7^ pfu were incubated with increasing quantities of anti-VSV-G or anti-HIV-1 Env Ab for 30 min at room temperature (RT). The Ab-bound complexes were collected by centrifugation and resuspended in 150 ul of 1%BSA-PBS containing 2 ug/ml of anti-human IgG Alexa555 or anti-mouse IgG2a Alexa555 (Becton Dickinson). Following incubation for 30 min at 37°C, the complexes were collected by centrifugation and resuspended in 150 ul of PBS. 30,000 particles were analyzed with a modified LSRII flow cytometer (Becton Dickinson) with forward scatter (FSC) and side scatter (SSC) set to log_10_ scale and threshold set to 4000. Data was analyzed using FlowJo software version 9.4 (Tree Star), where complexes were gated according to positivity compared to an alum only control. Median fluorescent intensity (MFI) was determined for each Ab dilution tested from which 4-parameter curves were fitted using Prism Version 6 (GraphPad).

As an alternative to analysis of alum-bound virus particles, flow cytometry was conducted with solutions of virus particles bound by Abs 10^9^ pfu of purified rVSV-EnvG_4_-G_6_^IN^ or parental rVSV-G_4_ vector suspended in PBS were incubated with 20 uM SYTO 63 membrane permeable dye (Life Technologies) to stain nucleic acids. Stained virus was centrifuged in PBS-1%BSA for 45 min at 35,000 g at 4°C using a SW36 rotor (Beckman Coulter). Virus pellets were resuspended 1 ug/ml anti-VSV-G^IN^ (Vi10) in PBS-1%BSA for 30 min at RT after which virus-Ab complexes were collected by centrifugation and resuspended in PBS-1%BSA containing 2 ug/ml of anti-mouse IgG2a Alexa555 and incubated for 30 min at RT. Virus was collected again by centrifugation and resuspended in PBS, and then analyzed by flow cytometry as described above. Minimum threshold settings on SSC were employed to increase sensitivity for small particles and FSC and SSC parameters were set to log_10_ scale as previously described [60]. Deionized water was run for 15 min to equilibrate for low threshold noise. ∼50,000 events were acquired for the PBS control, while ∼10^6^ events were acquired for the virus samples.

### Mouse studies

#### Ethics statement

Female Balb/c mice were purchased from the Jackson Laboratory and maintained at SUNY Downstate Medical Center in a facility accredited by the Association for Assessment and Accreditation of Laboratory Animal Care International (AAALACI) that also had been inspected by the USDA. The SUNY Downstate Institutional Animal Care and Use Committee approved the study protocols (Permit numbers 11-10005, 11-10272, and 14-10409). Anesthesia was carried out by inhalant Isoflurane (3%–5%) or intraperitoneal injection of 60 mg/kg Ketamine + 0.5 mg/kg Dexdomitor followed by 25 ug/kg Antisedan as reversal agent. Depth of anesthesia was confirmed by a lack of response to hind limb toe pinch (withdrawal reflex) and a subjective decrease in respiratory rate. Normothermia was maintained with a warm water-circulated heating pad. Mice were placed in a clean "recovery cage" after procedures were complete on sterile gauze where they were observed until they regained the righting reflex. Animals were then returned to a clean home cage of 4 mice per cage (conventional microisolator cage using Sanichip bedding). Cages were kept at 64–79°F, mice were fed a Purina 5001 rodent diet, and received environmental enrichment (treats and huts with wheels). All euthanasia was performed by intraperitoneal injection of (1 ml/10lbs) Euthasol. All efforts were made to minimize suffering.

#### Virus replication

Prior to the study, microchips (BMDS IPTT-300 Transponder, BioMedic Data Systems, Seaford, DE) were implanted in 48 mice under inhalant anesthesia to facilitate temperature monitoring. At day 0, 12 6-week old mice per test group received an intranasal (IN) inoculation under injected anesthesia of 20 ul PBS only, or PBS containing 10^7^ PFU of rVSV-G_4_, rVSV-EnvG_4_-G_6_, or rVSV-EnvG_4_-ΔG. On day 1, 4 mice from each group (16 total) were euthanized. A terminal blood collection via cardiac puncture was obtained from anesthetized animals followed by post mortem perfusion with PBS and removal of the brain, nasal turbinates, lungs, and spleen. Mice inoculated with rVSV-G_4_ (rVSV with no gene insert and the natural gene order) became noticeably ill by day 2, experiencing extreme weight loss and decreased temperature These mice were given 2 cc of Normosol subcutaneously, placed on a heating pad to help increase body temperatures, and fed high caloric gel, but these interventions appeared to have little effect. Two of these mice died naturally on day 2 of the experiment, and their tissues were not further analyzed. Four of the remaining mice in the rVSV-G_4_ group were euthanized on day 3 as scheduled along with 4 mice from each of the other study groups, and tissues were collected as described above. The remaining 2 mice from the rVSV-G_4_ group were euthanized on day 4 and not used in the study. On day 7 all remaining mice were euthanized as scheduled and tissues were collected as above.

#### Tissue sample preparation

Fresh mouse tissue specimens were weighed, suspended in 4°C phosphate-buffered saline containing sucrose phosphate buffer with glutamate (0.2 M sucrose, 7.0 mM K_2_HPO_4_, 3.8 mM KH_2_PO_4_, 5.0 mM glutamic acid) (PBS/SPBG) to 10% weight/volume (*w/v*), and homogenized using an Omni THQ – Digital Tissue Homogenizer (Omni, Kennesaw, GA) with plastic autoclavable probes. To avoid cross contamination among tissue specimens, each tissue was homogenized with a separate probe. Homogenates were clarified by centrifugation and supernatants were frozen at −80°C in 100 µl aliquots for subsequent RNA extraction.

#### RNA extraction, cDNA synthesis, and Real-time quantitative PCR

Viral nucleic acid was extracted from clarified homogenized tissues using reagents from the RNeasy Mini kit (Qiagen, Valencia, CA) as described previously [61], [62]. Subsequent cDNA synthesis to detect VSV genomic RNA (gRNA, negative sense) was performed using a Sensiscript Reverse Transcriptase kit (Qiagen) as described [61]. To specifically detect VSV N mRNA, the final reactions contained 10 µM of the VSV-specific mRNA RT-primer that was substituted for the VSV N forward primer. Otherwise, the reagents and the conditions for the reverse transcription (RT) step remained the same [61]. To ensure that free VSV-specific mRNA RT-primer did not carry over to the qPCR step, exonuclease 1 (2 U/sample) (New England BioLabs, Ipswich, MA) was added at the end of the cDNA reaction. qPCR was carried out with reagents from the QuantiTect Multiplex PCR Kit (Qiagen) using methods previously described [61], [62]. Amplification and detection were performed with a Stratagene Mx3005P Sequence Detection System. All samples were tested in duplicate. The detection limit of the qPCR assay was 9.0×10^1^ copies/mg of tissue or 500 copies/mL of blood.

#### Immunogenicity study

Twenty-eight mice were used at 6–8 weeks old for each of 2 identical immunogenicity studies, of which one study is described here in detail. On day 0 of each immunogenicity study, pEnvG/pIL-12 was administered by electroporation to 12 anesthetized mice (Groups 1–3, see Table 1). Mice were shaved over the left tibialis anterior muscle and 30 µg of pEnvG +25 µg of pIL-12 were delivered by intramuscular (IM) electroporation (EP) with the Ichor TriGrid System (Ichor Medical Systems, Inc. San Diego, CA) in a total volume of 20 µL PBS. At week 3, Groups 1-3 received another pEnvG/pIL–12 administration as above, while an additional 8 mice (Groups 4–5) were inoculated IN with rVSV-EnvG_4_-G_6_ as described for the replication study above, and 8 mice (Groups 6–7) were anesthetized by injection and inoculated with 10^7^ PFU rVSV-EnvG_4_-G_6_ in PBS by 40 µL IM injection (20 µL into each quadriceps muscle. At week 6, Group 1 (4 mice) received pEnvG/pIL-12 by IM EP as above, Groups 2, 5, and 6 received an IM rVSV injection as above, and Groups 3, 4, and 7 received an IN rVSV inoculation as above. At day 0, week 3, week 6, and week 8, blood collection was performed on all mice while they were sedated as described above. At week 11 all mice were euthanized as described above and terminal blood collection and removal of spleen and lungs was performed post mortem.

**Table 1:** Murine Immunization Regimens.

Group (N = 4)	Prime*	Boost∧
1	pEnvG + pIL-12	pEnvG + pIL-12
2	pEnvG + pIL-12	**IM** rVSV-EnvG_4_-G_6_^IN^
3	pEnvG + pIL-12	**IN** rVSV-EnvG_4_-G_6_^IN^
4	**IN** rVSV-EnvG_4_-G_6_^IN^	**IN** rVSV-EnvG_4_-G_6_^NJ^
5	**IN** rVSV-EnvG_4_-G_6_^IN^	**IM** rVSV-EnvG_4_-G_6_^NJ^
6	**IM** rVSV-EnvG_4_-G_6_^IN^	**IM** rVSV-EnvG_4_-G_6_^NJ^
7	**IM** rVSV-EnvG_4_-G_6_^IN^	**IN** rVSV-EnvG_4_-G_6_^NJ^

* Groups 1–3 were primed twice, at weeks 0 and 3; groups 4–7 were primed once at week 3.

∧All groups were boosted at week 6. **IN**: Intranasal; **IM**: Intramuscular. *Superscript* IN/NJ denotes strain of rVSV used.

#### Serum antibody assays

ELISA was used to assess serum antibody titers. 100 ng of D7324 (anti-HIV-1 Env gp120 C5) (Aalto) was coated in 50 ul of PBS to Polystyrene, flat-bottom, high-binding, ½ area plates (Corning, Corning, NY) and incubated at 4°C overnight using a procedure similar to described previously [63]. The plates were washed in PBS containing 0.05% Tween-20 (0.5% PBS-T buffer) and blocked with 3% bovine serum albumin (BSA; Sigma) in PBS for 1 h at 37°C. 50 ng of purified JR-FL gp140 foldon trimer protein was subsequently added to each well assays were performed as described previously using either Horseradish Peroxidase (HRP) goat anti-mouse IgG (total IgG; Biolegend, San Diego, CA), or biotin goat anti-mouse IgG1or IgG2a (Jackson ImmunoResearch, West Grove, PA) followed by streptavidin-HRP [64], [65]. All inter-group comparisons were analyzed by Mann-Whitney test using Prism Version 5.04 (GraphPad).

Week 11 serum from selected study groups was subjected to IgG purification. 50 µl of sera from pairs of mice was combined (i.e., 2 pairs per group) and IgG was extracted using 0.2 mL NAb Protein G Spin Columns (Thermo Scientific), according to manufacturer's protocol. Extracts were tested for protein concentration, and subjected to SDS-PAGE and western blot as described above to ensure sample purity/quality. A standard TZM-bl neutralization assay was performed as previously described, wherein 7.2 ug/mL purified IgG was titrated 4-fold, combined with an equal volume of HIV-1 pseudo-typed with SF162.LS or SIVmac239 (negative control) Env, and incubated 1 h at 37°C prior to the addition of TZM-bl cells [66].

#### T cell assays

To analyze T cell responses, 1.5×10^6^ leukocytes isolated from the spleen or lungs were incubated with 2 µg/ml anti-CD28 (BD Biosciences) and 20 µg/ml JR-FL gp140 foldon trimer for 2 hrs at 37°C, before incubation with 10 µg/ml brefeldin A (Sigma) for 5 hrs at 37°C. Cells were stained for viability with LIVE/DEAD Fixable Violet Dead Cell Stain (Molecular Probes, Eugene, OR) before surface staining for CD4 and CD8 (BD Biosciences, San Jose, CA). Cells were subsequently fixed and permeabilized with Cytofix/Cytoperm solution (BD Biosciences) before treatment with an intracellular stain for IFNγ, IL-2, and TNFα (BD Biosciences). Cells were resuspended in 0.5% paraformaldehyde and were analyzed with a modified BD LSR II flow cytometer. Data and statistical analysis was done using FlowJo software version 9.4 (Tree Star) and SPICE version 5.22 (Mann-Whitney test) [67].

## Results

### rVSV-EnvG_4_-G_6_ and pEnvG characterization

In order to develop a genetically stable, replication-competent rVSV vector that expressed membrane-anchored EnvG abundantly and exposed the immune system to trimers incorporated in the infected cell plasma membrane and in the envelope of progeny virus particles, we investigated modifications of both the vector and insert. For the purpose of increasing Env expression and incorporation into virus particles relative to G, the G_IN_ or G_NJ_ gene was moved to the 5′ terminus of the genome (the 6^th^ position, Fig. 1) to reduce G mRNA synthesis and the Env coding sequence was inserted in the 4^th^ position normally occupied by G. This rVSV-EnvG_4_-G_6_ vector strategy was chosen for several reasons. First, translocation of the G gene is a stable modification that will not be subject to genetic reversion. Second, shuffling the position of the G gene is known to modulate VSV replication, and its placement in the least transcribed region of the genome is expected to be an attenuating mutation that will reduce risk associated with using a live VSV-based vaccine [18], [38], [68]. Third, we postulated that reduction of G expression would lessen the immunodominance of G [68]. Finally, and perhaps most important, we wanted to use a vector design approach that minimally disturbed the very potent VSV transcription apparatus and the rVSV-EnvG_4_-G_6_ configuration maintained a nearly native arrangement of the N, P, M, and L genes (Fig. 1).

The Env immunogen used in these studies was derived from a subtype B HIV-1 (strain JR-FL). In addition to swapping the Env CT for the shorter G^IN^ CT to produce an Env-G hybrid (EnvG) similar to those previously described, we also substituted the Env transmembrane (TM) domain with sequence from G^IN^, and replaced the SS with the VSV G SS or the CD5 SS [17], [36], [43], [69].

The EnvG gene inserts also required modification to generate genetically stable vectors. Stable vectors were generated when the EnvG nucleotide sequence was designed using a VSV codon bias and the gene optimization rules described in Methods, which included use of synonymous codons to disrupt homopolymeric sequences greater than 4 nucleotides. We found this approach greatly improved genetic stability, as over the course of virus rescue, plaque purification, and amplification of virus seed stocks, we observed that homopolymeric regions, particularly poly-A or poly-T, were more prone to nucleotide substitutions (data not shown). The following work was conducted with rVSV-EnvG_4_-G_6_^IN^ and rVSV-EnvG_4_-G_6_^NJ^ vectors whose EnvG inserts included all of the above modifications.

For the purpose of including a DNA prime as part of animal studies, the EnvG gene was also optimized with a mammalian codon bias, and cloned into a pCMV mammalian expression vector (pEnvG). To confirm EnvG expression, transfected 293T cell lysates were analyzed by western blot and bands were detected corresponding to gp120 and gp160 (data not shown).

The rVSV-EnvG_4_-G_6_ vectors were rescued and clonal isolates were prepared by plaque purification. EnvG, G, and VSV N expression were assessed by western blot using lysates prepared from infected Vero cells (Fig. 2a). Blots were probed with an anti-VSV G C-tail Ab, which recognized bands equivalent to EnvG gp41 and gp160, as well as a 60 kDa band corresponding to VSV G. Bands, which we labeled with the conventional gp41 and gp160 nomenclature, migrated somewhat faster than typically observed because the Env cytoplasmic tail has been replaced with the short 29-amino acid domain from VSV G. The results confirmed EnvG and G expression and indicated that the CD5 SS swap significantly increased the quantity of JR-FL EnvG observed in total cell lysates resulting in quantities that were equal or greater than G (Fig. 2a). As well, appropriate cleavage by cellular proteases of EnvG presursor into its gp120-like and gp41-like subunits was observed. A polyclonal antiserum specific for VSV N (at 45 kDa) also was used to provide a control for comparison of EnvG expression from the various vector constructs.

To evaluate incorporation of EnvG into rVSV-EnvG_4_-G_6_ particles, plaque-purified viruses were amplified in Vero cells and were then purified by ultracentrifugation in sucrose gradients. The purified viral stocks were confirmed to be composed primarily of viral proteins by conducting SDS-PAGE and Coomassie brilliant blue staining (data not shown). When western blotting was performed to analyze the relative quantities of the gp160 precursor and mature, proteolytically cleaved Env species, anti-gp120 antibody recognized both gp120 and gp160 as expected. Analysis by densitometry indicated that ∼35% of the reactive species was gp120 and ∼65% was gp160 in the rVSV-EnvG_4_-G_6_^IN^ particles (Fig. 2b). Though EnvG incorporation also was demonstrated in rVSV-EnvG_4_-G_6_^NJ^ particles, the quantities were considerably lower compared to the IN strain; therefore, the proportions of gp160 and gp120 could not be accurately determined (Fig. 2b). When a similar analysis of rVSV-EnvG_4_-G_6_^IN^ was conducted using anti-G^IN^ CT antibody, which recognized G and two species of EnvG (the gp160 precursor and the gp41 subunit), the relative quantities of gp41 to gp160 were ∼75% to 25%, indicating that the majority of the Env precursor had been cleaved. Furthermore, this analysis suggested that total EnvG incorporated in the virus particle (gp160 band + gp41 band) was ∼70% of the quantity of G (Fig. 2c). Finally, probing with an anti-G Ab cross-reactive for IN and NJ strains confirmed G incorporation for both rVSV types (Fig. 2d).

To measure the combined attenuating effect of EnvG gene insertion and the G gene shuffle on replication *in vitro*, rVSV-EnvG_4_-G_6_^IN^ and rVSV-EnvG_4_-G_6_^NJ^ propagation in Vero cells was compared to their unmodified rVSV-G_4_ progenitors. Cell monolayers were infected with 0.1 pfu per cell and medium supernatant was collected over a period of 51 hrs after which infectious virus released into the medium was quantified by plaque assay. The growth curves indicated that the infections proceeded with similar kinetics but that the rVSV-EnvG_4_-G_6_ viruses produced 5-22 fold less virus than their progenitors at most time points and never attained a peak titer equivalent to the unmodified rVSV (Fig. 2e).

To determine if the EnvG construct was capable of mediating membrane fusion and if the rVSV-EnvG_4_-G_6_ vector displayed functional EnvG trimers, various assays were conducted with CD4+CCR5+ GHOST cells as this cell line is susceptible to infection via G- or EnvG- mediated routes [53], [54]. Firstly, 293T cells transfected with either the pEnvG or empty vector were overlaid with CD4+CCR5+ GHOST cells 48 h after transfection. Forty-eight hours after overlay, cells were visualized by light microscope (Fig. 3a). It was evident in the pEnvG-transfected culture that large, multinucleated syncytia had formed that were not visible in the cultures transfected with empty vector. Additionally, CD4+CCR5+ GHOST cells were infected with parental rVSV-G_4_^IN^ or rVSV-EnvG_4_-G_6_^IN^ vectors which had been pre-incubated with VSV-G-specific nAb Vi10 and/or an HIV-1 Env-specific nAb cocktail, or in media alone (Fig. 3b). Cultures infected in the presence of nAb also were maintained post-infection in nAb-containing media. Eight hours post infection, cells were visualized and monolayers infected with rVSV-G_4_^IN^ exhibited notable cell death, which was exemplified by numerous rounded-up and floating cells as well as obvious gaps in the cell monolayer not visible in the uninfected controls (Fig. 3b, row 1, column 1). In comparison, cultures infected with rVSV-EnvG_4_-G_6_^IN^ were found to contain numerous ‘giant cells’ (syncytia) characteristic of Env-mediated cell fusion, as well as rounded-up cells and gaps in the cell monolayer (row 2, column 1). Incubation in the presence of Vi10 appeared to completely inhibit infection by the parental rVSV-G_4_^IN^ but not by rVSV-EnvG_4_-G_6_^IN^, as the cells exposed to the rVSV-G_4_^IN^-Vi10 mixture were indistinguishable from the uninfected controls (compare row 1, columns 1, 2 and 4) while cytopathic effect and giant cells were still evident in the rVSV-EnvG_4_-G_6_^IN^ culture, albeit reduced compared to infection without nAb (compare row 2, columns 1 and 2). As expected, incubation of rVSV-G_4_^IN^ in the presence of the anti-Env nAb cocktail had no effect on VSV infection; however, cytopathology was highly diminished in the rVSV-EnvG_4_-G_6_^IN^ culture, with only a few syncytia visible (compare row 2, columns 1 and 3). When incubated with both Vi10 and the Env-specific nAbs, infection of the CD4+CCR5+ GHOST cells by rVSV-EnvG_4_-G_6_^IN^ was completely inhibited (compare row 2, columns 1, 3, and 4).

### EnvG and G surface expression

**Figure 4:**
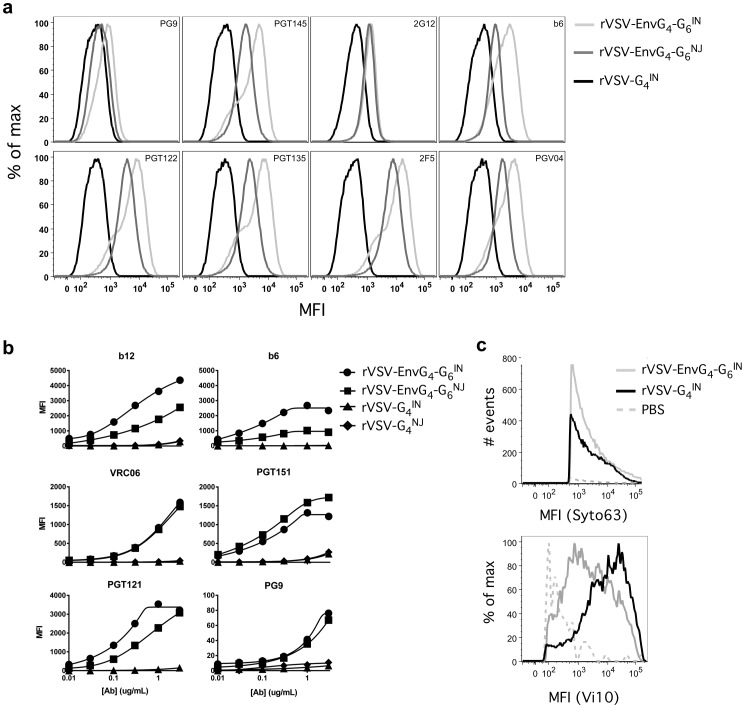
Surface staining of rVSV-EnvG_4_-G_6_-infected Vero cells and rVSV-EnvG_4_-G_6_ particles. (a) After a 22 hr infection, Vero cells were detached from plate by gentle trypsin treatment and resuspended in PBS. 5×10^6^ cells were analyzed for VSV G and HIV-1 EnvG surface expression. All cells analyzed for Env staining were first gated as positive for G staining. (b) For rVSV staining, 10^9^ pfu of virus was bound to alum at 37°C with agitation. rVSV/alum conjugates were stained with titrated anti-VSV-G (Vi10) or anti-HIV-1 Env Ab followed by anti-human IgG or anti-mouse IgG2a Alexa555 and acquired on a modified LSRII flow cytometer. Median fluorescent intensity (MFI) was determined for each Ab dilution. (c) 10^9^ pfu of virus was incubated with SYTO 63 nucleotide stain in PBS for 30 min at RT followed by incubation with anti-VSV-G (Vi10) and then anti-mouse IgG2a Alexa555. Virus was analyzed as described above. Minimum threshold settings on SSC were used to increase sensitivity for small particles and FSC and SSC parameters were set to log scale. Deionized water was run for 15 min to equilibrate for low threshold noise. ∼50,000 events were acquired for the PBS control, ∼10^6^ events were acquired for virus samples. Particles staining positive for nucleic acid that were above the noise threshold were gated on, and the amount of anti-G staining for those populations were compared.

G and EnvG incorporation into infected cell membranes and the envelope of rVSV-EnvG_4_-G_6_^IN^ and rVSV-EnvG_4_-G_6_^NJ^ was analyzed by multiparameter flow cytometry (Fig. 4a). Cells were stained with anti-VSV G that recognized the ectodomain of G^IN^ and G^NJ^ and a panel of anti-HIV-1 Env Abs. Analysis of infected cells showed that all cells positive for EnvG expression also were positive for G, indicative of VSV infection (data not shown). When cells were stained with anti-Env Abs, EnvG was detected on the cell surface and reacted with antibodies specific for structural features that included the CD4 binding site (b6, PGV04), the V2/glycan (PGT145, preferential binding with trimer), the V3/glycan (PGT122, PGT135), and the MPER region (2F5), while low-level recognition by PG9 (V2/glycan) was also observed (Fig. 4a) [33], [34], [56], [64], [70], [71]. Higher levels of Ab binding were generally observed for rVSV-EnvG_4_-G_6_^IN^ compared to rVSV-EnvG_4_-G_6_^NJ^ -infected cells, supporting the western blot data showing reduced expression levels of EnvG by the vector strain that coexpressed G^NJ^.

Purified virus particles adsorbed to alum also were stained with a panel of anti-Env Abs to examine the antigenicity of the EnvG incorporated in the virus particle (Fig. 4b). Among the CD4 binding site Abs tested, it was found that both viral strains bound b6 (prefers uncleaved Env) and b12 (recognizes cleaved and uncleaved Env), and VRC06 (exhibits strongly preferential binding to cleaved, trimeric Env) [72], [73]. Similarly, both viruses bound PGT151, which binds selectively to cleaved trimeric Env (gp120/gp41 interface of cleaved trimers; [74]). Both viruses were recognized by PGT121 (V3/glycan), and exhibited low-level binding to PG9 (Fig. 4b). Weak binding to PG9 is consistent with previous studies using Subtype B JR-FL Env [34]. Additionally, rVSV-EnvG_4_-G_6_^IN^ and rVSV-G_4_^IN^ particles were analyzed directly by flow cytometry after being co-stained with a nucleic acid dye (SYTO 63) and anti-G^IN^. Particles staining positive for nucleic acid that were above the SSC noise threshold were selected and the amount of anti-G^IN^ staining for those populations were compared. It was found that rVSV-EnvG_4_-G_6_^IN^ exhibited approximately a 1-log reduction in median fluorescence intensity (MFI) representing anti-G^IN^ staining compared to the rVSV-G_4_^IN^ parental virus (Fig. 4c).

### Replication and immunogenicity of rVSV-EnvG_4_-G_6_ vectors in mice

**Figure 5:**
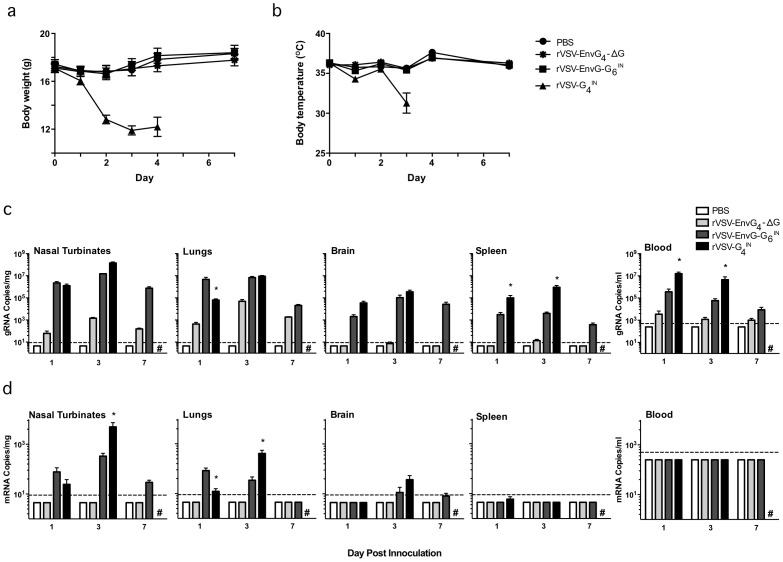
Effect of IN rVSV-EnvG_4_-G_6_ adminstration. (a) Mean body weights and temperatures (b) of inoculated mice. (c) Mean copy numbers of VSV N genomic RNA or (d) VSV N mRNA per mg of indicated tissue or mL of blood. N = 4–12, dependent on study day. Fresh tissue specimens were homogenized, clarified by centrifugation and supernatants were subjected to RNA extraction and qPCR. All samples were tested in duplicate. Dotted lines indicate limits of detection. SEM is shown. *p<0.05 for comparison of rVSV-EnvG_4_-G_6_ to rVSV-G_4_. All PBS and rVSV-EnvG_4_-ΔG values were found to be significantly lower than rVSV-EnvG_4_-G_6_ and rVSV-G_4_ values.

The *in vivo* replication of rVSV-EnvG_4_-G_6_^IN^ at days 1, 3, and 7 post-IN administration was examined and compared to that of parental rVSV-G_4_^IN^ as well as to a chimeric virus vector that encodes functional EnvG but lacks the G gene (rVSV-EnvG_4_-ΔG) and is therefore incapable of replication in mice (manuscript in preparation). Weights and body temperatures were monitored daily and the data showed that mice inoculated with rVSV-EnvG_4_-G_6_^IN^ experienced transient, minimal losses in body weight (6% maximum loss) within 2 days post administration that were not significantly different than those experienced by the mice inoculated with rVSV-EnvG_4_-ΔG or the PBS-inoculated control mice (Fig. 5a). Body temperatures followed a similar trend in that no significant differences were observed among mice administered PBS, rVSV-EnvG_4_-G_6_^IN^, or rVSV-EnvG_4_-ΔG (Fig. 5b). In contrast, mice inoculated with rVSV-G_4_^IN^ experienced severe weight loss up to a maximum of 35% of their pre-inoculation body weights by day 3 and appeared to lose the ability to maintain normal (36.5°C–38.0°C) body temperature (mean day 3 body temperature, 31°C). Therefore, all mice receiving rVSV-G_4_^IN^ were euthanized by day 4 of the experiment.

rVSV-EnvG_4_-G_6_^IN^ genomic RNA (gRNA) was detected at days 1, 3, and 7 (where applicable) in all tissues tested and in the blood (Fig. 5c). The highest quantities of gRNA were in the respiratory tract, i.e., the nasal turbinates and lungs, with similar copy numbers detected for rVSV-G_4_^IN^ and rVSV-EnvG_4_-G_6_^IN^ groups at days 1 and 3; however, on day 1 rVSV-EnvG_4_-G_6_^IN^-inoculated mice were found to have significantly higher levels of gRNA in the lungs. gRNA also was detected in the nasal turbinates and lungs of mice inoculated with the non-replicative ΔG virus, though the quantity was significantly (1–4 log) lower compared to the replication-competent strains. At day 7, gRNA quantities in the respiratory tract remained fairly constant for rVSV-EnvG_4_-G_6_^IN^ and remained above the amounts detected for the nonreplicating ΔG virus. As mentioned above, animals infected with the parental rVSV-EnvG_4_ were euthanized before the day 7 timepoint. In the spleen and blood, significantly lower quantities of gRNA were found in mice infected with rVSV-EnvG_4_-G_6_^IN^ compared to those infected with rVSV-G_4_^IN^ at days 1 and 3 (p = 0.014). In whole brain, gRNA was detected at days 1 and 3 in animals infected with either rVSV-EnvG_4_-G_6_^IN^ or rVSV-G_4_^IN^ and also at day 7 in mice infected with rVSV-EnvG_4_-G_6_^IN^. Day 7 gRNA differences could not be assessed as all rVSV-G_4_^IN^ animals were euthanized by day 4. No gRNA was detected in PBS-inoculated mice.

When VSV N mRNA was quantified to provide an indicator of the replication activity in the different tissues, the highest amounts were detected in the respiratory tract, with significantly greater levels (∼1log difference, p = 0.014) found in mice inoculated with rVSV-G_4_^IN^ compared to those inoculated with rVSV-EnvG_4_-G_6_^IN^ at day 3. Mice inoculated with rVSV-EnvG_4_-G_6_^IN^ were found to have significantly higher levels of mRNA in the lungs at day 1 (Fig. 5d). No mRNA was detected in the blood above background. Only 1 rVSV-EnvG_4_-G_6_^IN^-inoculated animal had detectable levels of mRNA in the brain (<50 copies/mg) compared to 3 out of 4 for the rVSV-G_4_^IN^ group (<100 copies/mg). As expected, no mRNA was detected in the host-restricted rVSV-EnvG_4_-ΔG- or PBS-inoculated mice.

The immunogenicity of the rVSV-EnvG_4_-G_6_ vectors in 6–8 week old mice was analyzed after vaccination by IN and IM routes using 4 different prime/boost regimens (IN/IN, IM/IM, IN/IM, and IM/IN, see Table 1 for regimen detail). In some groups, the effect of a pEnvG DNA prime delivered by IM electroporation (EP) and adjuvanted by co-delivery of a plasmid encoding Interlukin-12 (pIL-12) also was investigated, as recently we demonstrated control of SIVmac239 replication in RMs immunized IM by EP SIV DNA + pIL-12 followed by SIV-recombinant Adenovirus 5 (rAd5) boost [75]. Mice primed with DNA (Groups 1–3) were vaccinated 2 times by EP at 0 and 3 weeks after which Group 1 was boosted with a 3^rd^ dose of DNA and Groups 2 and 3 were boosted with rVSV-EnvG_4_-G_6_^IN^. Mice primed with viral vector (Groups 4–7) were vaccinated once either IM or IN with rVSV-EnvG_4_-G_6_^IN^ at week 3 and were then boosted at week 6 with rVSV-EnvG_4_-G_6_^NJ^ (IN or IM), which encodes the NJ serotype G [76]. As found for IN administration, no adverse effects were observed after IM vaccination.

**Figure 6:**
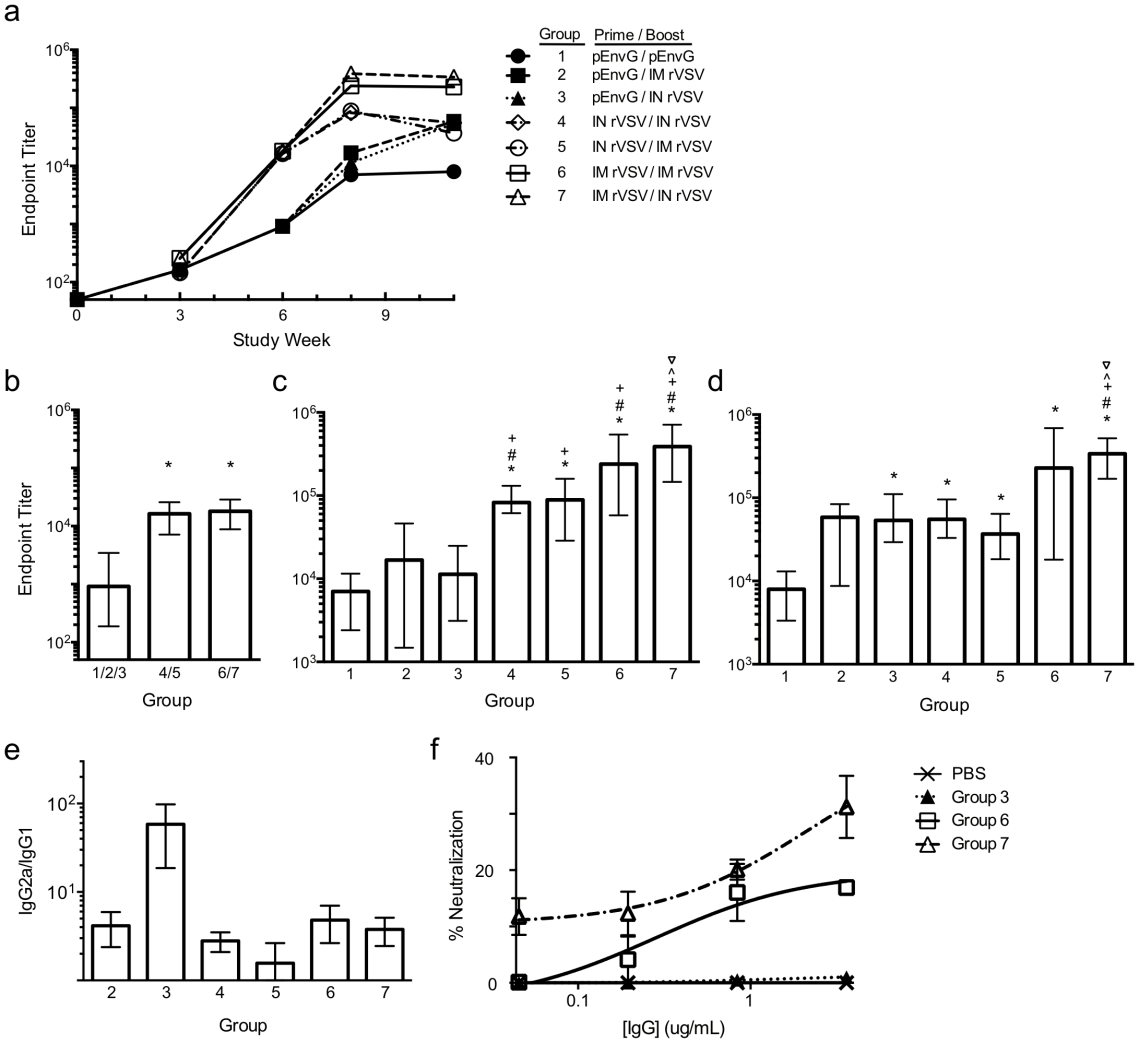
Serum anti-Env IgG antibody responses. (a) Mean anti-Env endpoint titers (4 animals/group) against JR-FL foldon trimer were determined over the course of the 11 week experiment as described previously.(b) Week 6 endpoint titers for mice primed with pEnvG/pIL-12 (group 1/2/3, N = 12), IN rVSV-EnvG_4_-G_6_ (group 4/5, N = 8), or IM rVSV-EnvG_4_-G_6_ (group 6/7, N = 8). *p<0.05 compared to group 1/2/3. (c) Week 8 endpoint titers for all groups. (d) Week 11 endpoint titers for all groups. *p<0.05 compared to group 1. ^#^p<0.05 compared to group 2. ^+^p<0.05 compared to group 3. ∧p<0.05 compared to group 4. ^∇^p<0.05 compared to group 5. (e) Ratio of IgG2a to IgG1endpoint titers at week 11. (f) Neutralization of HIV-1 virus pseudo-typed with SF162.LS Env as measured in a standard TZM-bl neutralization assay using IgG purified from week 11 sera of selected groups. SEM is shown.

Env ELISA endpoint titers were monitored over the course of the study (Fig. 6a). Serum from mice primed at weeks 0 and 3 with pEnvG/pIL-12 (Groups 1–3) had a mean Env-specific ELISA endpoint titer of 915 at week 6, which was significantly lower than the titers of mice vaccinated once at week 3 with rVSV-EnvG_4_-G_6_^IN^ by the IN or IM route (Groups 5–7; Fig. 6b, p<0.0001). No difference in Env-specific titers was observed at week 6 between mice primed IN or IM with VSV-EnvG_4_-G_6_^IN^ (mean endpoint titer, 17197).

A boost was given at week 6, and animals were immunized either with a 3^rd^ dose of pEnvG/pIL-12 (Group 1) or a rVSV-EnvG_4_-G_6_ vector administered IN or IM (Groups 2–7). Two weeks post-boost (week 8, Fig. 6c), all rVSV-primed regimens (Groups 4–7) were found to elicit significantly higher Env-specific Ab titers than vaccination with pEnvG/pIL-12 only (Group 1; 12-55-fold difference; p = 0.0143), and the pEnvG/pIL-12 – IN rVSV regimen (Group 3; 7-34-fold difference; p = 0.014). Group 4–7 titers were also 5-23-fold higher than the pEnvG/pIL-12 – IM rVSV regimen, though these differences were not significant. Intriguingly, at this time point neither pEnvG/pIL-12 – rVSV regimen was shown to induce significantly greater Ab responses than those in animals vaccinated 3 times with pEnvG/pIL-12, and titers for the pEnvG/pIL-12 prime groups were no higher than those elicited by a single IN or IM rVSV immunization as recorded at week 6 (Fig. 6a). Furthermore, among the groups that were both primed and boosted with rVSV, the IM/IN and IM/IM regimens elicited the highest Env-specific titers (mean, 389169 and 239101, respectively). The IM/IN-induced titers were significantly greater than those elicited by the IN/IN and IN/IM routes (∼4-fold difference; 0.029≥p≥0.014).

Five weeks post-boost (week 11, Fig. 6d), Env-specific titers remained virtually unchanged, with the exception of the pEnvG/pIL-12 – IN rVSV group (Group 3), in which titers increased 5-fold compared to week 8 (p = 0.014). Though the week 11 mean titer for the pEnvG/pIL-12 – IM rVSV boost group (Group 2) was also higher compared to week 8, this difference was not significant. As well, mean titers for the groups primed with pEnvG/pIL-12 were still not significantly higher than those elicited by one rVSV immunization as measured at week 6 (Fig. 6a). Ultimately, we found that IM rVSV-EnvG_4_-G_6_^IN^ prime combined with IN rVSV-EnvG_4_-G_6_^NJ^ boost (Group 7) elicited the highest Env-specific serum antibody titers at week 11 (mean, 338525), which were significantly greater than all other regimens (8-42-fold difference; p = 0.014) except rVSV IM/IM.

At week 11, selected sera were analyzed for isotype-specific titers and the presence of HIV-1-specific nAb. IgG1- and IgG2a-specific ELISA was performed on sera from groups 2–7. It was found that all groups exhibited an IgG2a-skewed phenotype, most exhibiting titers 1.5 – 4 – fold higher compared to those specific for IgG1 (Fig. 6e). This phenotype was particularly striking for the pEnvG/pIL-12 – IN rVSV group (Group 3), whose IgG2a titers were ∼60-fold greater than those specific for IgG1 (p = 0.057 compared to all other groups).

Sera from groups 3, 6, and 7 were also subjected to IgG purification and analyzed for neutralizing activities in the TZM-bl pseudo-typed HIV-1 neutralization assay. While IgG purified from the pEnvG/pIL-12 – IN rVSV group (Group 3) exhibited no more neutralizing activity against Clade B pseudo-typed virus (SF162.LS strain) than IgG purified from PBS- immunized mice, IgG from the rVSV IM/rVSV IM and rVSV IM/rVSV IN groups exhibited 17% and 31% neutralization of SF162.LS, respectively, at 3.6ug/mL (Fig. 6f). This difference in neutralization potency was not considered significant as it was below the 50% benchmark for the assay. Neutralization decreased as a function of titrated IgG, persisting at 12% neutralization for the rVSV IM/rVSV IN group (Group 7) at the lowest concentration of IgG tested (∼45 ng/mL). No neutralization of the control pseudo-typed virus (SIVmac239) was evident at these IgG concentrations, though low-levels of non-specific activity was present at high concentrations of IgG (>15 ug/mL IgG; data not shown).

**Figure 7:**
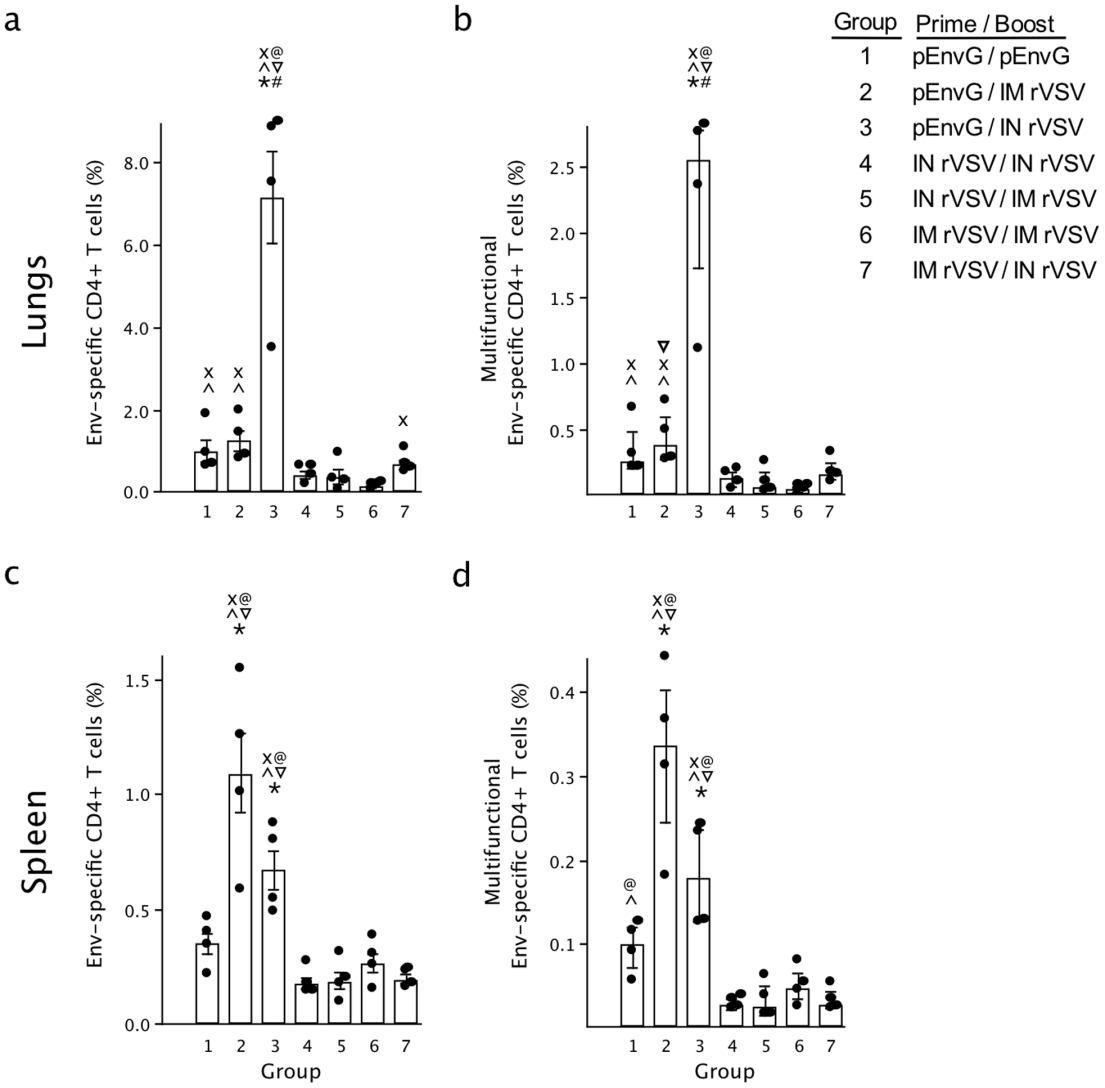
Frequency of JR-FL Env-specific CD4+ T cell responses in the lung and spleen. 1.5×10^6^ leukocytes isolated from the lungs (a, b) andspleen (c, d) at the end of the study were stimulated *ex vivo* with JR-FL gp140, anti-CD28 and brefeldin A before being analyzed by flow cytometry for production of IFNg, IL-2 and TNFa. The total frequency of cytokine secreting CD4+ T cells (%) producing IFNγ, IL-2 or TNFα are shown on the left (a, c) and the frequency of multifunctional CD4+ T cells producing any combination of IFN γ +, IL-2+, and/or TNFα+ on the right (b, d). *p<0.05 compared to group 1. ^#^p<0.05 compared to group 2. ∧p<0.05 compared to group 4. ^∇^p<0.05 compared to group 5. ^@^p<0.05 compared to group 6. ^x^p<0.05 compared to group 7. Bars represent the median (with SEM), individual animals are shown.

Additionally, Env-specific T cell responses at week 11 were evaluated using cells harvested fromin spleens and lungs. Cultured lymphocytes were stimulated with Env protein after which intracellular cytokine staining was performed for IFNγ, IL-2, and/or TNFα. In the lungs, we found that the frequency of Env-specific CD4+ T cells was significantly higher in mice vaccinated with pEnvG/pIL-12 prime - IN rVSV boost (Group 3) compared to all other regimens (7.2% frequency, 6–70-fold difference, p = 0.042; Fig. 7a). pEnvG/pIL-12 alone (Group 1) and pEnvG/pIL-12 prime - IM rVSV boost (Group 2) induced similar frequencies of Env-specific CD4+ cells compared to the IN/IN and IM/IM rVSV prime - rVSV boost combinations (Groups 4 and 6; p = 0.042) (Fig 7a). Among the rVSV-primed groups (Groups 4–7), no significant differences in the frequency of Env-specific CD4+ T cells were observed, and only the IN/IN and IM/IN regimens (Groups 4 and 7) elicited significantly more CD4+ T cells compared to the PBS controls (p = 0.042; Fig. 7a and data not shown). Notably, CD8+ Env-specific T cells in the lung were very low and not significantly higher for any group compared to the PBS controls (data not shown). One measure of T cell immune response quality is the production of any combination of IFNγ, IL-2 or TNFα in response to specific antigen stimulation at the single cell level. Multifunctional T cells are capable of producing a combination of cytokines and of producing more of each cytokine on a per cell basis [77]. Upon analysis of the quality of the CD4+ T cell response in the lungs it was observed that the Group 3 response was comprised of a significantly higher frequency of double- and triple-positive multifunctional CD4+ T cells (secreting either IFNγ, IL-2, and TNFα, or IFNγ and TNFα) compared to all other groups (2.5% frequency, 6–50-fold difference, p = 0.042; Fig. 7b).

In the spleen, it was found that both pEnvG/pIL-12 prime - rVSV boost groups elicited the highest frequencies of Env-specific CD4+ T cells (0.7%–1.1%), which were significantly greater than all other regimens (1.5–5-fold difference, p = 0.042; Fig. 7c). No significant differences in the frequency of Env-specific CD4+ T cells among the rVSV-primed regimens were observed, though all elicited significantly more specific CD4+ T cells than the PBS controls (10–70-fold difference, p = 0.042, data not shown). The pEnvG/pIL-12 prime - rVSV boost groups also elicited significantly more multifunctional CD4+ T cells than all rVSV-primed groups (0.27% frequency; 3–10-fold difference, p = 0.042; Fig. 7d), though the pEnvG/pIL-12 prime – IN rVSV boost regimen did not elicit more multifunctional CD4+ T cells in the spleen than pEnvG/pIL-12 prime – pEnvG/pIL-12 boost. As found in the spleen, specific CD8+ T cells were very low and not significantly higher for any group compared to the PBS control (data not shown).

## Discussion

We developed a replication-competent, attenuated, immunogenic rVSV vector platform that expressesfunctional, membrane-anchored HIV-1 Env trimers that are incorporated in the cell plasma membrane as well as progeny virus particles. Importantly, the EnvG immunogen encoded by the vector is fusogenic and is able to support rVSV replication in CD4+/CCR5+ GHOST cells when G is blocked by nAb (Fig. 3). Previous studies on rVSV surface protein expression have shown the Env CT to be inhibitory, while the VSV G CT can induce more efficient transit in the secretory system and incorporation of Env into VSV particles [16], [17], [36], [78], [79]. Therefore, our clade B EnvG was designed with a VSV G CT, and we also substituted the Env TM domain with that of VSV G as we found in other studies with similar vectors that this improved surface expression and vector propagation (data not shown). As illustrated by Figures 2-4, rVSV-EnvG_4_-G_6_^IN^ particles contained significant quantities of EnvG. Possibly having both the VSV G TM and CT increased EnvG localization at sites of VSV budding, as TM domains have been shown to be important to the localization of proteins within lipid rafts [80], [81]. As G and EnvG were co-displayed on these viruses, sharing a common TM domain may also have contributed to overall ability of the vector to propagate to high titers, remain genetically stable during scale-up, and incorporate significant quantities of both glycoproteins into the VSV particle. Interestingly, the rVSV-EnvG_4_-G_6_^NJ^ vector did not incorporate EnvG as effectively (Fig. 2 and 4). This may have been due to the mismatch of the G^IN^-derived TM and CT domains, as differences in G^IN^ and G^NJ^ postranslational processing have been documented and these strains share only ∼50% sequence identity [40], [82], [83].

Western blot analysis conducted with Vero cells infected with rVSV-EnvG_4_-G_6_^IN^ (C5 SS) indicated that the quantities of EnvG in total cell lysates after a 24 hr infection to be roughly equivalent to that of G, or perhaps even greater when the relative amounts of G and both the gp160 and gp41 bands are considered (Fig. 2a). This level of EnvG expression appears higher than that observed for the prototypical rVSV-G_4_-EnvG_5_ vectors shown to be protective against SHIV infection in RMs, for which EnvG expression in cell lysate was estimated to be 31% that of G [17], [28]. Furthermore, our analysis suggested that total EnvG incorporation into rVSV-EnvG_4_-G_6_^IN^ to be ∼70% of that of G, with incorporation of furin-cleaved EnvG to be ∼54% that of G. This relative level of EnvG incorporation is estimated to be considerably higher than that observed for rVSV-G_4_-EnvG_5_ vector, whose cleaved EnvG incorporation was previously estimated to be ∼3% of the level of G [17]. This improvement was potentially due to effects of one or more elements of the vector and immunogen designs we used, including placement of EnvG in the 4^th^ position and/or G in the 6^th^ position, the inclusion of the VSV TM domain, VSV-specific codon optimization, and/or the use of a CD5 SS.

Probing for EnvG by western blot using an Ab recognizing gp120 estimated the proportion of cleaved (i.e. functional) EnvG on the surface of rVSV-EnvG_4_-G_6_^IN^ particles to be roughly 34%. As densitometry analysis using an anti-G CT Ab, which employs the gp41 band to measure cleavage, estimated the proportion of cleaved EnvG to be more than 2x greater, it is plausible that a proportion of the cleaved EnvG incorporated in the viral particles are gp41 ‘stumps’. This may have been in part due to dissociation of gp120 from gp41 during virus purification [84]. Nevertheless, the mixture of EnvG forms on the viral surface is consistent with previous observations that a notable proportion of Env on HIV-1 viruses is non-functional, including cleaved monomers, gp41 monomeric and oligomeric stumps, and uncleaved gp160 oligomers [84]. It widely accepted that only a minority of Env on the surface of HIV-1 viruses is mature trimer, and that gp160 cleavage is essential to the proper folding of Env, which leads to functionality and appropriate recognition by neutralizing Abs [73], [84]–[87]. Recognition of rVSV-EnvG_4_-G_6_ particles and infected cells by Abs that preferentially bind trimers such as PGT151, VRC06, and/or PGT145, and the ability of this virus to infect CD4+CCR5+ GHOST cells in the presence of a completely neutralizing concentration of anti-G Ab demonstrates that this vector can produce and display EnvG in a functional, trimeric conformation [33], [72]–[74].

Translocation of G to the 6^th^ genetic position of the VSV genome was expected to be an attenuating mutation [18]. Compared to prototypical rVSV-G_4_-EnvG_5_ vectors, whose safety profile was considered unacceptable for a first-in-man clinical trial with a replicating VSV vector [18], [23], [28], [31], [32], [76], [88], initial work with G_6_ vectors encoding Gag indicated that this vector design significantly increased the virus dose required to reach an intracranial LD50 endpoint in mice [38]. Our *in-vitro* replication data with vectors encoding EnvG showed that there was a 5-20-fold growth attenuation compared to rVSV-G_4_ in Vero cells (Fig. 2e) indicating that addition of the EnvG gene and downregulation of G expression had a significant effect on replicative capacity. Consistent with the interpretation that G downregulation played a role in growth attenuation, flow cytometry data indicated that there was a ∼1 log reduction in G incorporation in virus particles compared to the rVSV-G_4_ progenitor (Fig. 4c). Attenuated replication *in vivo* also was evident in viral N mRNA levels that were ∼1 log lower for rVSV-EnvG_4_-G_6_ compared to the parental rVSV-G_4_ at the predominant sites of viral replication (nasal turbinates and lungs) on day 3 post inoculation. Interestingly, in nasal turbinate and lung tissues at day 1, there were significantly higher mRNA and gRNA quantities in mice infected with the rVSV-EnvG_4_-G_6_ vector. The elevated quantities of the rVSV-EnvG_4_-G_6_ vector at day 1 (about 24 hours post-infection) might indicate that the rVSV-EnvG_4_-G_6_ inoculum might have a higher particle-to-pfu ratio.

Importantly, rVSV-EnvG_4_-G_6_-inoculated mice experienced no observed illness following IN inoculation, no more weight loss than observed in the PBS controls, and no signs of distress. This contrasted sharply with the morbidity and mortality observed in animals that were inoculated the progenitor vector rVSV-G_4_ (Fig.5) or the results observed in earlier studies with the VSV-Gag_4_-G_5_ prototype VSV-HIV vector [24] It well known that mice are highly susceptible to VSV neuroinvasion when they are experimentally infected by IN administration or by other routes, and that susceptibility is age-dependent with young mice being prone to encephalitis; thus, rodent models have been used to study VSV neurovirulence and have been used to evaluate potential risk of using VSV vectors in [62], [89]–[91]. It also is known that considerable vector engineering is needed to generate genetically stable vectors that exhibit substantially reduced neurovirlence in the murine model, and that it is very difficult to completely eliminate the appearance of viral nucleic acid in the brain particularly after IN inoculation even with modified VSV that has been attenuated sufficiently to prevent symptoms [62]. Consistent with these earlier findings, rVSV-EnvG_4_-G_6_ gRNA was detectable in whole brain extracts prepared from mice vaccinated by the IN route, but it is important to note that N mRNA quantities were just above the limit of detection in only 1 of 4 animals indicating that gene expression and replication in the brain was quite restricted, which was consistent with our observation of no significant weight loss, absence of fever, and no distress caused by rVSV-EnvG_4_-G_6_ vaccination. These results indicate that IN vaccination with rVSV-EnvG_4_-G_6_ vectors does not cause typical neurovirulence in mice and that viral replication in the brain is significantly attenuated resulting in the absence of detectable neurological symptoms. The rVSV-EnvG_4_-G_6_ vector has the potential to be advanced as a safe vaccine platform, but additional studies will be required to determine the anatomical location of the viral genomes in the brain and whether their presence is associated with virus propagation in those locations.

Our rVSV-EnvG_4_-G_6_ vectors were designed to stimulate potent Env-specific humoral and cellular immune responses, as protective immunity to HIV-1 infection will undoubtedly require both. In the current study we have demonstrated that B and T cell responses can be stimulated in mice using this vector. A single IM or IN administration of rVSV-EnvG_4_-G_6_ induced equivalently potent humoral responses. It was evident that an IM (systemic) prime followed by a mucosal (IN) boost (Group 7) had the most potent humoral effect, as significantly higher Ab titers were observed for this regimen (Fig. 6). The benefits of a heterologous vaccination site approach have been noted previously, and will be further tested in future NHP studies [92]. The IM/IN regimen also exhibited notable IgG-mediated, Env-directed neutralizing activity, extending the notion that a proportion of EnvG incorporated into rVSV-EnvG_4_-G_6_ particles and produced by infected cells is conformationally intact and therefore can induce nAbs. Additionally, the magnitude of all Env-specific Ab responses were ∼2–3 logs greater than those previously observed in mice using rVSV-G_4_-EnvG_5_ vectors, which elicited titers of <800 even after heterologous boost [88], [93]. This increase in specific Ab relative to earlier studies in mice could be the result of the relatively higher amounts of EnvG on rVSV-EnvG_4_-G_6_ particles and infected cells.

*Env-specific CD4+ T cells also were elicited, particularly when rVSV was primed 2x with pEnvG/pIL-12, which increased the frequency of these cells as much as 70-fold, dependent on the immunization route and site of T cell measurement. This effect of a DNA/pIL-12 prime supports previous studies that demonstrate an enhancement by pIL-12 on CD4+ T cell quantity and quality (multifunctionality), with little to no effect on CD8+ T cells, and a potentially negative effect on Env-specific Ab [94]–[98]. Furthermore, the effects of pIL-12 are dose-, antigen-, and likely species-dependent, and though we selected a dose shown to be beneficial for T cell stimulation, a lower dose may have been more beneficial overall [94]–[96]. The positive effect on CD4 T cell stimulation of the pEnvG/pIL-12 prime was particularly notable in the lungs after the rVSV IN boost (Group 3). This was likely due to the considerable vascularization of the respiratory mucosa, which results in the highly efficient delivery of antigen to local and recruited antigen presenting cells in the lungs, and induces a robust T cell response to the local respiratory rVSV infection, where rVSV replication was almost exclusively apparent (nasal turbinates and lungs, Fig. 5c) [25], [28], [99], [100]. Indeed, the rVSV has been extensively demonstrated to elicit strong immune responses upon IN administration due to its mucosal tropism [28], [76], [100], [101]. As expected from the strong impact on CD4 T cells, IgG isotype analysis revealed that the pEnvG/pIL-12 –prime, rVSV IN boost group exhibited the most significant Th1-skewed humoral response, with an IgG2a/IgG1 ratio that was 15-40-fold greater than those observed for the other regimens and indicative of the IFNγ-dominant cytokine environment induced by the robust CD4 T cell response in the lung typical of VSV infection [102]–[104].

In conclusion, the study presented herein describes our novel rVSV-G_6_ vector that displays relatively large amounts of a unique HIV-1 EnvG antigen that is fusogenic, functional for virus replication, and capable of inducing potent Env-specific immune responses, including nAbs. The replication of this vector is attenuated in mice compared to an unmodified rVSV, and elicits no pathology after IN or IM administration. We are confident it can be used safely in NHPs and currently are advancing a candidate HIV vaccine encoding clade A BG505 trimers in rhesus macaques [58].
